# Acute Stress Impacts Executive‐Social Function: Evidence From Prefrontal Activation and fNIRS‐Based Hyperscanning

**DOI:** 10.1002/brb3.71214

**Published:** 2026-01-15

**Authors:** Zhihua Guo, Yue Gong, Liu Yang, Yushan Li, Rui Qiu, Xia Zhu

**Affiliations:** ^1^ Department of Military Medical Psychology The Fourth Military Medical University Xi'an China; ^2^ National Translational Science Center for Molecular Medicine & Department of Cell Biology The Fourth Military Medical University Xi'an China

**Keywords:** acute stress, compensatory mechanism, dyadic cooperation, executive function, hyperscanning, interbrain synchronization, prefrontal cortex

## Abstract

**Background:**

Acute stress has complex effects on executive function and social behavior; however, the direction of these effects is inconsistent across studies, and the underlying neural mechanisms remain poorly understood. This study investigated the behavioral and neural effects of acute stress on executive function and dyadic cooperation and their relationships.

**Methods:**

Eighty‐six healthy male undergraduates (18–25 years) were randomly assigned to stress (*n* = 44; Trier Social Stress Test for Groups [TSST‐G]) or control groups (*n* = 42; placebo TSST‐G). The participants completed executive function tasks (3‐back, Go/Nogo, Stroop, and task‐switching) and cooperative button‐pressing tasks pre‐ and postintervention, with a counterbalanced order. Functional near‐infrared spectroscopy (fNIRS) was performed simultaneously.

**Results:**

Stress impaired the practice‐induced improvement in 3‐back accuracy observed in the control group, although it did not significantly affect other performance metrics. During the 3‐back, Stroop, task‐switching, and cooperative tasks, increased and decreased prefrontal cortex (PFC) activation from baseline to postintervention were observed in the stress and control groups, respectively. Furthermore, greater bilateral dorsolateral PFC (DLPFC) interbrain synchronization (IBS) changes during the cooperative task were observed in the stress group. Cognitive flexibility and cooperation were positively linked both behaviorally and neurally.

**Conclusions:**

TSST‐G‐induced stress disrupted the learning‐related enhancement of working memory; however, response inhibition, interference control, cognitive flexibility, and cooperative performance were preserved. The concurrent observation of trends toward increased neural activation and IBS under stress is compatible with, but does not prove, potential compensatory mechanisms. The identified neural and behavioral correlations point to a potential connection between executive and social processes under stress. We tentatively frame these exploratory observations within the “Executive–Social Function Coupling Hypothesis” as a heuristic model for future research. The implications of these preliminary findings are discussed.

## Graphical abstract

Trends in increased PFC activation and increased IBS may be indicative of compensatory neural mechanisms preserving performance under stress. Cognitive flexibility correlated with cooperative outcomes, tentatively supporting a neurobehavioral executive‐social link during acute stress inviting more research.

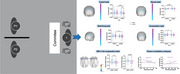

## 1 Introduction

Acute stress, such as stress associated with natural disasters, family crises, and even significant events such as important exams, is common in daily life (Ying et al. 2022; Yan et al. 2024). Acute stress refers to the nonspecific responses (including physiological, psychological, behavioral, and neurological responses) exhibited by individuals when they face real or perceived temporary challenges to their ability to maintain internal homeostasis (Folkman and Lazarus 1986; Girotti et al. 2024; Spencer et al. 2024). Subjectively, acute stress significantly increases negative emotions (such as anxiety) and decreases positive emotions (Zhao et al. 2022; Liang et al. 2024). Physiologically, acute stress results in the activation of the autonomic nervous system (ANS), primarily the sympathetic–adrenomedullary (SAM) axis, triggering the secretion of catecholamines (e.g., norepinephrine). Furthermore, acute stress activates the hypothalamic‒pituitary‒adrenal (HPA) axis, leading to the release of glucocorticoids (primarily cortisol in humans). Together, these mechanisms influence an individual's neural and physiological activities (Ulrich‐Lai and Herman 2009; Berretz et al. 2021; O'Connor et al. 2021; Geissler et al. 2023).

Executive function, also known as executive control, involves various higher order cognitive processes associated with the prefrontal cortex (PFC) that enable successful goal‐directed behavior. The core components of executive function include inhibition control (response inhibition and interference control), working memory, and cognitive flexibility (Diamond 2013). The effects of acute stress on executive function remain controversial. Acute stress may impair (Schoofs et al. 2008; Lieberman et al. 2016; Santos‐Carrasco and De la Casa 2024), have no effect on (Stone et al. 2021; E. Deuter et al. 2024), or even enhance working memory performance (Schoofs et al. 2013; Liang et al. 2024). Furthermore, few studies have investigated cognitive flexibility, but the existing studies have reported mixed results, including reports of stress‐induced increases (Lin et al. 2018; Morava et al. 2023; Knöbel et al. 2024), decreases (Kalia et al. 2018; Marko and Riecansky 2018), or no effect on cognitive flexibility (Miguez 2019). In terms of inhibition control, response inhibition is predominantly observed to increase following stress (Schwabe et al. 2013; Dierolf et al. 2018; Spencer et al. 2024), with some studies reporting no effect or decreased response inhibition following stress (Jiang and Rau 2017a; Kan et al. 2021). Moreover, although few studies on interference control have been performed, conflicting findings have been reported (including both increases (Knöbel et al. 2024; Yan et al. 2024) and decreases (Kan et al. 2021; Ma et al. 2022) following stress). Although a 2016 meta‐analysis (Shields, Sazma, et al. 2016) reported that acute stress impairs working memory, cognitive flexibility, and interference control while enhancing response inhibition, the considerable heterogeneity across studies highlights the need for more research. One confounding variable accounting for the heterogeneity is sex (Schoofs et al. 2013; Shields, Trainor, et al. 2016; Zandara et al. 2016; Kalia et al. 2018). In this study, we restricted enrollment to only male participants to address the documented moderating effects of sex on acute stress.

Executive function plays a crucial role in social interactions (Decety et al. 2004; Qiao et al. 2025; Zhang et al. 2025). It underlies multiple processes, such as the maintenance of overarching goals, generation of flexible behavior, inhibition of inappropriate responses, and ongoing monitoring. All of these processes are integral to successful social interactions. Cooperation, one of the fundamental forms of social cognition and social interactions, refers to the actions or intentions through which individuals or groups collaborate to achieve common goals or mutually beneficial outcomes (Decety et al. 2004). In recent decades, the effects of stress on social cognition and behaviors have been widely explored. The most prototypical male stress response manifests as the “fight‐or‐flight” pattern (Cannon 1932). Conversely, females may adopt the “tend‐and‐befriend” biobehavioral response pattern when they encounter stressors (Taylor et al. 2000; Taylor 2006). These patterns imply that females under stress tend to exhibit increased cooperative and prosocial behaviors, whereas males may exhibit increased aggression and decreased prosocial behaviors (Vinkers et al. 2013; Sollberger et al. 2016; Nickels et al. 2017; Youssef et al. 2018; Potts et al. 2019; Wekenborg et al. 2022). However, recent studies suggest that males can also exhibit a “tend‐and‐befriend” response pattern (von Dawans et al. 2012; Wolf et al. 2015; Berger et al. 2016; Tomova et al. 2020), leading to varying findings. The extent to which prosocial behaviors in males under acute stress are increased or weakened remains unclear. Furthermore, no prior studies have examined ecologically valid dyadic cooperation among males under acute stress beyond the use of traditional single‐brain experimental paradigms, such as economic games and decision‐making tasks.

One theory explaining the neural mechanisms underlying the effects of acute stress on executive function is the biphasic–reciprocal model. According to this model, acute stress triggers a reallocation of neural resources toward the salience network (centered on the amygdala) while reducing the responses available to the dorsolateral PFC (DLPFC)‐dominated executive control network (ECN) (Hermans et al. 2014); this model is supported by several studies (Arnsten 2009; Qin et al. 2009; Hermans et al. 2011; Arnsten 2015). However, exposure to psychosocial stressors has also been shown to elicit increased activity in the ECN, particularly the DLPFC (Porcelli et al. 2008; Rosenbaum et al. 2018; Meier and Schwabe 2024). Hence, more research is needed to determine how acute stress affects the neurological activities underlying executive function.

Existing research has yet to systematically examine the neural mechanisms through which acute stress influences cooperative behavior, particularly among male participants. Hyperscanning technology, which simultaneously measures brain activity in two or more individuals during social interactions (Czeszumski et al. 2020; Carollo and Esposito 2024), represents a promising approach to address this gap. Among neuroimaging techniques such as electroencephalography (EEG), functional magnetic resonance imaging (fMRI), and functional near‐infrared spectroscopy (fNIRS), fNIRS is particularly well‐suited for investigating cooperative behavior due to its advantages in portability, ease of use, relatively high temporal resolution, robustness to motion artifacts, and suitability for naturalistic experimental settings (Carollo and Esposito 2024; Luo et al. 2025). In general contexts (not acute stress), fNIRS‐based hyperscanning studies have revealed that interbrain synchronization (IBS) in the PFC (Czeszumski et al. 2022), especially in the DLPFC (Cheng et al. 2015; Reindl et al. 2018; Hu et al. 2021), plays a critical role in interpersonal cooperation. One fNIRS hyperscanning study investigating the effects of acute psychosocial stress on interpersonal cooperation in young women revealed a steady improvement in cooperative behaviors with more widespread IBS in the PFC, indicating that acute stress strengthens the female “tend‐and‐befriend” response in cooperation (Zhang et al. 2021). However, whether a similar manifestation exists in males under acute stress remains unclear.

Researchers have elucidated the involvement of ECNs in dyadic cooperative behavior (Zhang et al. 2020, 2024). A synthesis of existing literature indicates that the functional brain networks involved in cooperation partially overlap with the neural foundations of executive function and, to some extent, share neural substrates with stress‐sensitive systems. Moreover, numerous studies have speculated that executive function processes are extensively involved during cooperative tasks (Cui et al. 2012; Jiao et al. 2024; Song et al. 2024; Zhang et al. 2024). For example, Zhang et al. (2024) found that collaborative cooperation elicited the engagement of the left DLPFC, proposing that this process relies on cognitive flexibility. However, the empirical validation of these frequently speculated links between executive function and cooperative behavior within a systematic framework is still lacking.

Notable gaps are present in the existing research. First, inconsistent findings regarding the behavioral and neural effects of acute stress on executive function and dyadic cooperation remain unresolved; second, the potential links between these processes under acute stress warrant preliminary exploration. In an attempt to address these research gaps, we designed this study to investigate the effects of acute stress on executive function and dyadic cooperation at both the behavioral and neural levels among male participants. Given the inconsistency in prior findings, our hypotheses were framed as exploratory: (1) On the basis of some evidence (Shields, Sazma, et al. 2016; Zhang et al. 2021), one possibility is that acute stress may impair working memory, cognitive flexibility, and interference control, while potentially increasing response inhibition and inducing a “tend‐and‐befriend” response in males; (2) stress‐related alterations in DLPFC activity during these processes are expected, consistent with the biphasic–reciprocal model; and (3) we will exploratorily assess whether specific executive subcomponents (e.g., cognitive flexibility) correlate with cooperative behavior under stress, and whether the neural activity of executive function and cooperation correlates. To our knowledge, this work represents the initial investigation into this topic. Given its fundamentally exploratory nature, the findings should be interpreted as preliminary.

## 2 Materials and Methods

### 2.1 Participants

Eighty‐six healthy male college students (mean age = 20.28 ± 1.56 years, range = 18–25 years) participated in this study. The participants were recruited from universities in Xi'an, China, through advertisements posted on university bulletin boards and social media platforms. This study exclusively recruited male participants to minimize potential confounding effects arising from the influences of the female menstrual cycle and oral contraceptives on the cortisol response to stress (Kirschbaum et al. 1999), as well as the aforementioned sex‐specific effects of stress on executive function and biobehavioral patterns. The inclusion criteria were as follows: (1) male sex; (2) right‐handedness; (3) normal or corrected‐to‐normal vision; (4) aged 18–25 years; (5) body mass index (BMI) between 18.5 and 28 kg/m^2^; and (6) Beck Depression Inventory‐II (BDI‐II) total score <14 to exclude potential confounding effects of depression on stress responses (Beck et al. 1996; Qi and Gao 2020). The exclusion criteria were as follows: (1) any history of smoking or alcohol consumption; (2) acute diseases such as acute appendicitis; (3) current use of any form of medication (e.g., hormonal or psychoactive medications); (4) a history of any neuropsychiatric disorders; and (5) severe physical conditions such as cardiovascular disease. The participants did not know the purpose of the study and were randomly assigned to two groups: the stress (*n* = 44, 22 dyads) and the control (*n* = 42, 21 dyads). To minimize the influence of preexisting social relationships on neural synchrony (Song et al. 2024), all dyads were composed of two unacquainted individuals. This was confirmed through pre‐experiment inquiry by the researchers. Participants within each dyad were randomly paired and explicitly instructed not to interact with each other during the experiment to avoid the formation of interpersonal rapport. The participants were asked to complete an online survey with questionnaires assessing basic demographic data and traits to analyze individual differences. G^∗^Power 3.1.9.6 was used to compute the a priori sample size, and a minimum sample size *N* of 34 (17 per group) was needed, with a medium effect size of *f* = 0.25, a power of 1 − *β* = 0.80, and an *α* value of 0.05 (Cohen 1992; Faul et al. 2007). Even at the dyadic level (i.e., pairs consisting of two individuals), the sample size in this study was adequate. The participants provided written informed consent before the experiment. The study was approved by the Xijing Hospital Ethics Committee of the Fourth Military Medical University (KY20252173‐F‐1) and was performed according to the Declaration of Helsinki. After completing the experiment, the participants received monetary compensation for their time.

### 2.2 Study Design and Procedure

**FIGURE 1:**
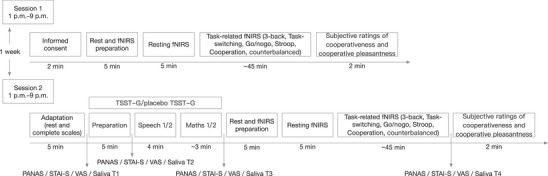
Schematic illustration of the study design. fNIRS, functional near‐infrared spectroscopy; PANAS, positive and negative affect scale; STAI‐S, state version of the State‐Trait Anxiety Inventory; TSST‐G, Trier Social Stress Test for Groups; VAS, visual analogue scale.

We used a randomized, parallel‐group, and stress‐controlled design with time (baseline vs. posttest) as the within‐subject factor and group (stress vs. control) as the between‐subject factor. The timeline of the study design is shown in Figure 1. The participants underwent two experimental sessions separated by at least 1 week. To control for diurnal variations in cortisol secretion, each session started between 13:00 and 21:00, following a procedure adopted from prior research (Hensel et al. 2022). The start times of all experimental sessions were counterbalanced across the stress and control groups to ensure no systematic bias due to diurnal variation in cortisol levels. In the first session, the dyads came to the fNIRS laboratory room and provided informed consent; then, they underwent a 5‐min resting‐state fNIRS recording (the results are not included in the present analysis). The dyads subsequently completed baseline behavioral tasks, with fNIRS data recorded during the respective tasks. The tasks were counterbalanced across and within groups. Upon the completion of all the tasks, the participants rated their attitudes toward their partners and subjective experiences during the cooperation task (e.g., “I liked the partner I was paired with during the task” and “I maintained high concentration throughout the cooperation task”) on a 5‐point Likert scale from 1 (strongly disagree) to 5 (strongly agree). Participants within a dyad were separated by a baffle and seated side by side in front of separate computer screens and keyboards. They were asked to refrain from verbal and physical interaction throughout the entire experiment. One day before the second session, the participants were asked to obtain sufficient sleep and avoid alcohol, coffee, and vigorous physical activities. In addition, they were asked to not ingest beverages or food other than water 1 h before the appointment (Labuschagne et al. 2019). In the second session, in Room A, the dyads first rinsed their mouths and completed some scales to assess potential confounding variables, including personality traits such as trait anxiety, cooperative personality, rumination, empathetic concern, and group preference. This period served as the rest and adaptation period. Then, the modified Trier Social Stress Test for Groups (TSST‐G) and the corresponding control test (placebo TSST‐G) were performed in Room B. Next, the resting‐state fNIRS data were collected for 5 min in the fNIRS laboratory room (i.e., Room C). The dyads subsequently completed behavioral tasks identical to those administered in the first session (with order counterbalanced across and within groups) concurrent with the fNIRS recording. After the tasks were completed, the subjective ratings of the participants were also collected. Questionnaires assessing emotional changes were administered at the T1, T2, T3, and T4 time points in Room A, and salivary samples were collected to measure changes in cortisol levels. At the end of the experiment, the participants were debriefed about the purpose of the study and were paid for their participation.

### 2.3 Executive Function Tasks

The participants completed four counterbalanced cognitive tasks assessing executive function (Guo et al. 2022; Guo, Qiu, et al. 2023; Lu et al. 2024): working memory (3‐back task), inhibition control (Go/Nogo and Stroop tasks), and cognitive flexibility (task‐switching paradigm). To reliably capture task‐related hemodynamic responses with fNIRS, a well‐established block design was implemented across all the tasks. Although individual trials were relatively brief, the block structure allowed for the aggregation of multiple trials within each condition, thereby increasing the signal‐to‐noise ratio and enabling a robust estimation of the hemodynamic response. This approach is consistent with prior fNIRS studies (Nagashima et al. 2014; Guo et al. 2022). Although trials in the 3‐back task were presented in a fixed order, those in the remaining tasks were randomized.

#### 2.3.1 3‐Back Task

**FIGURE 2:**
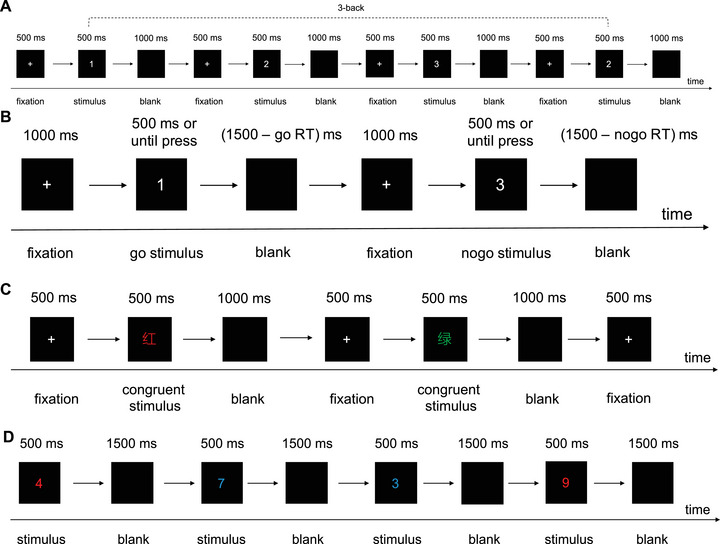
Cognitive tasks were performed to assess executive functions: (A) 3‐back task, (B) Go/Nogo task, (C) Stroop task, and (D) task‐switching paradigm. RT, response time.

For the 3‐back task (Figure 2A), a number stimulus ranging from 1 to 9 appeared on the screen in each trial, and the participants were instructed to press the “J” key when the target was identical to the number presented three numbers previously; otherwise, they pressed the “F” key. The formal test included 4 blocks, each containing 18 trials (2 s/trial), with a 30‐s rest period preceding each block.

#### 2.3.2 Go/Nogo Task

The Go/Nogo task was used to assess response inhibition ability (Figure 2B). In the Go/Nogo task, Go and Nogo blocks were alternated (three repetitions each, 12 trials per block, 2.5 s/trial), with a 30‐s rest period preceding each block. In the Go blocks, all 12 trials were Go stimuli; in the Nogo blocks, the Go and Nogo stimuli occurred equally (50% of each type of stimulus). The participants were instructed to press the “J” key in response to the Go stimuli (numbers 1, 2, and 4) and provide no response to the Nogo stimulus (number 3).

#### 2.3.3 Color–Word Stroop Task

The Color–Word Stroop task was used to measure interference control ability (Figure 2C). In this task, neutral, congruent, and incongruent blocks were alternated (two repetitions each, 18 trials per block, 2 s/trial), with a 30‐s rest period preceding each block. A neutral stimulus (“X”)or Chinese character (“红” for red, “绿” for green, and “黄” for yellow) printed in different colors (red, green, or yellow) was presented on the screen. In the neutral block, all the trials were neutral stimuli (different colors of “X”). During the congruent block, the character matched the color, whereas in the incongruent block, the character did not match the color. The participants were instructed to press “D,” “F,” or “J” on the keyboard when the color of the “X” or Chinese character was red, yellow, or green, respectively.

#### 2.3.4 Task‐Switching Paradigm

During each task‐switching trial (Figure 2D), a colored digit (0–9, excluding 5) was centrally displayed in either red or blue. For the red stimuli, the participants performed parity judgments by pressing “F” for odd numbers and “J” for even numbers. For the blue stimuli, a magnitude comparison with number 5 was performed: “F” for values <5 and “J” for values >5. The stimulus set contained numbers in four categories (odd numbers, even numbers, numbers greater than 5, and numbers less than 5) with equal proportions (25% each). The formal test included 4 blocks, each containing 20 trials (2 s/trial), with a 30‐s rest period preceding each block.

### 2.4 Cooperation Task

**FIGURE 3:**
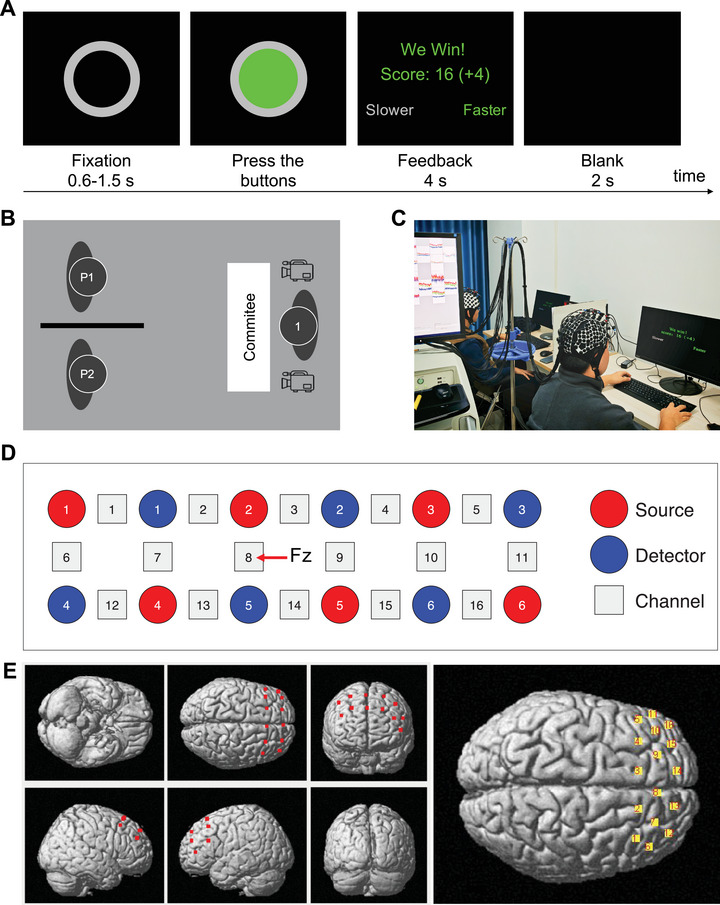
(A) Overview of the cooperation task. (B) Design of the stress exposure room. (C) A dyad engaging in the cooperation task in the experimental setup. (D) Arrangement of the optode probes for an individual in a dyadic setting. Channel 8 was located at Fz in accordance with the international 10–20 system. Emitter 2, channel 8, and detector 5 were aligned exactly along the sagittal reference curve. The distance between the emitter probes and detector probes was 3 cm. (E) The spatial anatomical locations of the optodes (left) and channels (right) were measured by a 3D digitizer with the virtual spatial registration method.

In this study, we employed a cooperation task paradigm identical to that used in our previous work (Lu et al. 2023). The task consisted of two blocks with a 30‐s rest period preceding each block. Each trial (20 per block) began with the presentation of a hollow grey circle at the center of the screen (Figure 3A). After a random 0.6–1.5 s delay, the circle turned green. The dyad members were given instructions that the green circle served as a “Go” signal, indicating that they should press their assigned buttons as synchronously as possible. The left‐seated participant pressed the “A” key, and the right‐seated participant pressed the “1” key on the numeric keypad. A simultaneous or near‐simultaneous key press was considered a successful cooperation and rewarded with four points, whereas a prolonged interval between presses was considered a failure and resulted in a deduction of four points. They were instructed to maximize the number of points earned. Successful cooperation was defined as a response time (RT) difference less than (RT1 + RT2)/8 (Cui et al. 2012; Lu et al. 2023), where RT1 and RT2 represent the RTs of the two participants. After both participants responded, a 4‐s feedback screen showed the trial outcome, numerical score, and which participant responded faster/slower through side‐matched text prompts to remind the participants to adjust their RTs. The next trial began after a 2‐s intertrial interval.

### 2.5 Stress Induction Task

A modified version of the validated TSST‐G was used to induce acute psychosocial stress in the dyads (von Dawans et al. 2011; Zhao et al. 2022; Lin et al. 2023). Participants in the stress group completed the TSST‐G, whereas the participants in the control group completed the placebo TSST‐G. The TSST‐G protocol included three sequential phases (Figure 1). Following a brief orientation, dyads were allocated a 5‐min preparation period. Each participant subsequently delivered a 2‐min introductory speech, simulating a competitive job interview scenario. The participants were separated by mobile dividing walls that restricted eye contact and social interaction. The interview committee consisted of a female experimenter in a white lab coat who was trained to withhold verbal and nonverbal feedback and was presented as an expert in the evaluation of nonverbal behavior. The use of a one‐member interview panel has been validated in previous studies (Pulopulos et al. 2020; De Smet et al. 2024; Spencer et al. 2024). The participants were informed that the TSST‐G session would be video‐recorded for performance evaluation purposes (Figure 3B). Immediately following the speech, dyads were instructed to perform an 80‐s serial subtraction task, alternating turns to subtract a predetermined two‐digit number from a four‐digit starting value. Each participant received an individual subtrahend and starting number to prevent learning effects. Once a mistake was made, the participant was required to restart from the initial number. The durations of the speech and arithmetic phases (2 min and 80 s, respectively) were set according to the established TSST‐G protocol (von Dawans et al. 2011). The placebo TSST‐G was designed in the same manner without exposure to any stressful components. After the preparation phase, the participants in the control group performed two tasks: reading a science text quietly and enumerating numbers. Neither the interview panel nor the digital cameras were present when the participants performed the tasks.

### 2.6 Data Acquisition

#### 2.6.1 Stress‐Related Data Acquisition

The acute stress induction protocol was validated by both physiological and psychological indicators in the present study. We used cortisol levels in saliva as a physiological indicator. Saliva samples were collected using Salivette sampling devices (Sarstedt, Germany). The participants kept the swabs in their mouths for 2 min without chewing and then placed them in a saliva collection device. All saliva samples were stored at −20°C. Consistent with validated stress research methodologies (Labuschagne et al. 2019; Zhen et al. 2021; Wang et al. 2023), the Positive and Negative Affect Scale (PANAS) (Huang et al. 2003), the state version of the State–Trait Anxiety Inventory (STAI‐S) (Spielberger et al. 1999), and the visual analogue scale (VAS) (Labuschagne et al. 2019) were used to evaluate the effects of stressors on emotional changes. Saliva samples and psychological measures were obtained at four time points (T1–T4; see Figure 1).

#### 2.6.2 fNIRS Data Acquisition

A 32‐channel LABNIRS fNIRS system (Shimadzu Co., Kyoto, Japan) with three wavelengths (780, 805, and 830 nm) was used to record the changes in the concentrations of oxygenated hemoglobin (HbO) and deoxygenated hemoglobin (HbR) (Figure 3C). One 2 × 6 optode probe patch with 16 measurement channels (6 source and 6 detector) was placed on each participant's PFC. The raw sampling period was 0.021 s. The source‒detector distance was set at 3 cm. Channel 8 was placed at the Fz point according to the international 10–20 system to ensure consistent optode placement across participants (Figure 3D). To determine the anatomical locations of the optodes and channels, we used a 3D digitizer (Fastrak, Polhemus, Colchester, VT, USA) to capture the coordinates of the optode positions on the basis of head landmarks (nasion, Cz, and left and right preauricular points) in real‐world space and registered the coordinates of the channels and optodes according to the standard Montreal Neurological Institute (MNI) template using the software package NIRS‐SPM (Figure 3E) (Singh et al. 2005; Ye et al. 2009). The virtual spatial registration method was applied to assess the consistency between the fNIRS channels and the anatomical structural labels in the Brodmann areas (Singh et al. 2005). The locations of all the channels are shown in Table 1. Channels located in the DLPFC were defined as regions of interest (ROIs) for subsequent analysis.

### 2.7 Data Analysis

#### 2.7.1 Salivary Cortisol Data Analysis

Cortisol concentrations were quantified using enzyme‐linked immunosorbent assay (ELISA) conducted by Quanzhou Ruixin Biotechnology Co. Ltd. (China). The intra‐ and inter‐assay variation coefficients were less than 15%. The area under the curve with respect to increase (AUCi) was calculated to assess the temporal increase in cortisol, as this measure adjusts for individual baseline differences (AUCi) (Pruessner et al. 2003).

#### 2.7.2 Behavioral Data Analysis

Working memory (3‐back task) was assessed on the basis of the response accuracy (primary outcome) and the mean RT for correct responses. Response inhibition (Go/Nogo task) was measured using the inverse efficiency score (IES, primary), goRT (mean RT in the correct Go trials), and Nogo accuracy (proportion of trials in which successful inhibition was demonstrated). The IES was calculated as the goRT divided by the overall accuracy (total correct trials/total number of trials) (Guo et al. 2022). The Stroop effect (incongruent RT minus congruent RT) and switching cost (switching RT minus repeated RT) were used to quantify the interference control capability (Stroop task) and cognitive flexibility (task‐switching paradigm). Higher 3‐back accuracy and lower IES, Stroop effect, and switching cost metrics indicated better performance. Dyadic cooperation was evaluated by the number of wins (primary), the absolute value of the average RT difference, and the sum of the average RTs of the two participants (Cui et al. 2012; Lu et al. 2023). Regarding the 3‐back task, 13 participants were excluded from the analysis because their accuracy at the baseline or posttest was zero (indicating a fundamental failure to understand or engage with the task's core “3‐back” updating rule), resulting in a final analytical sample of 36 participants in the stress group and 37 participants in the control group. For the Go/Nogo task, three participants were excluded because the participants were nonresponsive in certain blocks, yielding final samples of 41 participants in the stress group and 42 participants in the control group. For the Stroop task, two participants were excluded because their Stroop effect metric exceeded ±3 SDs from the mean, resulting in the inclusion of 43 (stress) and 41 (control) participants. All participants were included in the analyses for the task‐switching and cooperation tasks.

#### 2.7.3 fNIRS Data Analysis

Due to the absence of temporal markers required for fNIRS data analysis, one dyad from the stress group for the Stroop task and one dyad from the control group for the cooperation task were further excluded following the initial exclusion of participants based on their behavioral performance. We focused only on changes in the concentration of HbO because HbO is more sensitive to changes in cerebral blood flow than HbR is (Hoshi 2003; Zhang et al. 2023).

The activation analysis was conducted using the NIRS‐KIT MATLAB toolbox (Hou et al. 2021). After reviewing the data and ensuring data quality, preprocessing was performed, including detrending, motion correction via the temporal derivative distribution repair method (Fishburn et al. 2019), and bandpass filtering. For the cooperation task, a wider bandpass filter (0.01–0.1 Hz) (Lu et al. 2023) was applied to retain potential signal variance associated with the rapid interactive dynamics of dyadic cooperation. For all other individual cognitive tasks, the conservative 0.01–0.08 Hz bandpass filter was used to capture the hemodynamic responses. After preprocessing, task‐related neural activation (beta values) was detected using a general linear model (GLM). The GLM was specified such that the observed hemodynamic signal was modeled as a linear combination of task‐related regressors and an error term. No additional covariates were included. The regressors of interest were constructed by convolving a canonical hemodynamic response function (HRF) with a boxcar function representing the onset and duration of each task block. The timing of these blocks was precisely defined by event markers embedded in the experimental program. All the analytical steps followed the default pipeline implemented in the NIRS‐KIT toolbox.

For the IBS analysis, we preprocessed the fNIRS data using the Homer2 MATLAB package (Huppert et al. 2009). The data were imported into Homer2 and visually inspected to exclude channels with severe signal artifacts. The preprocessing steps included (1) automatic channel pruning via the enPruneChannels function; (2) the conversion of optical intensity to optical density (OD) using hmrIntensity2OD; (3) motion artifact detection using hmrMotionArtifactByChannel and spline‐based correction via hmrMotionCorrectSpline (Scholkmann et al. 2010); and (4) the exclusion of stimuli overlapping with motion artifact timepoints using enStimRejection. Finally, the OD signals were transformed into relative changes in HbO and HbR concentrations. Notably, no filtering was applied during preprocessing. The preprocessed data were then analyzed using the wavelet transform coherence (WTC) MATLAB package to calculate the cross‐correlation of variations in the HbO signal time series data (i.e., IBS) between each pair of channels for each dyad pair (Grinsted et al. 2004). The frequency of interest ranged from 0.0781 to 0.3125 Hz (corresponding to periods of 3.2 to 12.8 s), which was selected on the basis of previous studies (Cui et al. 2012; Pan et al. 2017; Lu et al. 2023). For each dyad, 16 × 16 channel combinations were possible; however, in the statistical analyses, we focused on only the symmetric IBS (i.e., the IBS between homologous channel pairs for each dyad). The average task‐related IBS across two task blocks was used as the final outcome measure.

#### 2.7.4 Statistical Analysis

IBM SPSS (version 26.0) software was used to conduct the statistical analyses. For the Go/Nogo task, the activation results were analyzed by beta differences between the Nogo and Go blocks. Similarly, for the Stroop task, the results were analyzed using the beta differences between incongruent and congruent blocks. The normality of the data distribution was evaluated using the Shapiro‒Wilk test. The Mann‒Whitney *U* test or independent samples *t*‐test was used to compare basic characteristics and baseline task measures between groups. A one‐sample *t*‐test was used to compare the IBS values against zero. To assess baseline dyadic compatibility in response speed, independent samples *t*‐test was used to compare mean RTs between partners within each dyad during the cooperative task at baseline. The stress induction effect, behavioral task performance, and fNIRS outcomes were analyzed using a generalized estimating equation (GEE) approach with a full factorial design, with time as the within‐subject factor and group as the between‐subject factor. An identity link function and unstructured working correlation matrix were selected based on superior model fit statistics. Although our analysis focused on interpreting the interaction effects, both interaction effects and the main effect of time (which quantifies practice effects across groups) for behavioral task performance are reported. For significant interaction effects, post hoc comparisons were conducted with Bonferroni correction. To control for baseline differences in IBS, the change values (posttest minus baseline) were computed, and the results were analyzed using an independent samples *t*‐test. The associations between dyadic executive function performance (calculated as the mean of both participants’ behavioral outcomes) and primary cooperation performance (number of wins) were analyzed using Pearson correlation analysis. Similarly, in channels with significant brain activity changes, correlations between dyadic brain activation (i.e., the mean of both participants’ beta values) during executive function tasks and IBS during cooperation were also examined with Pearson correlation. Additionally, Pearson correlation was used to examine relationships between neural activity in significant channels and primary behavioral performance measures (e.g., number of wins and 3‐back accuracy). The false discovery rate (FDR) method was applied to correct for multiple comparisons in GEE models examining fNIRS channel data. Statistical significance was defined as *p* < 0.05 for all analyses.

## 3 Results

### 3.1 Basic Characteristics of the Participants

The basic characteristics of each group are shown in Table 2, with statistics computed at both the individual and dyadic levels. The results revealed no significant differences between the stress and control groups in terms of age, BMI, years of education, trait anxiety, depression, cooperative personality, perceived stress levels, ruminative thinking, group preference, theory of mind, perspective taking, empathic concern, or trustworthiness (all *p*s > 0.05), at either level of analysis.

**TABLE 1:** Montreal Neurological Institute (MNI) coordinates and location information of all channels.

Channel	Brodmann area	MNI coordinates			Percentage
		*X*	*Y*	*Z*	
1	BA8—includes frontal eye fields	32	20	62	0.86
2	BA8—includes frontal eye fields	14	22	67	0.62
3	BA8—includes frontal eye fields	−11	21	68	0.58
4	BA8—includes frontal eye fields	−30	20	61	0.82
5	BA9—dorsolateral prefrontal cortex	−48	19	48	0.70
6	BA9—dorsolateral prefrontal cortex	41	31	48	0.92
7	BA8—includes frontal eye fields	22	33	59	0.89
8	BA8—includes frontal eye fields	1	34	57	1
9	BA8—includes frontal eye fields	−20	31	60	0.95
10	BA9—dorsolateral prefrontal cortex	−40	30	48	0.90
11	BA45—pars triangularis Broca's area	−54	26	29	0.58
12	BA9—dorsolateral prefrontal cortex	31	45	45	0.99
13	BA9—dorsolateral prefrontal cortex	12	47	53	0.72
14	BA9—dorsolateral prefrontal cortex	−11	45	52	0.73
15	BA9—dorsolateral prefrontal cortex	−29	42	45	0.98
16	BA45—pars triangularis Broca's area	−47	40	29	0.85

*Note*: The percentage indicates the extent to which each channel belongs to the corresponding cortical area.

**TABLE 2:** Basic characteristics of the groups.

Variable	Individual level				Dyadic level			
	Stress (*n* = 44)	Control (*n* = 42)	*t/U*	*p*	Stress (*n* = 22)	Control (*n* = 21)	*t/U*	*p*
Age (years)	20 (19, 21)	21 (19, 22)	737.5^a^	0.101	20.05 ± 1.35	20.52 ± 1.36	−1.158^b^	0.254
BMI (kg/m^2^)	22.57 ± 1.94	22.63 ± 1.9	−0.146^b^	0.884	22.57 ± 1.12	22.63 ± 1.32	−0.163^b^	0.871
Education (years)	14 (13, 16)	15 (13.75, 16)	776.5^a^	0.191	14.34 ± 1.35	14.74 ± 1.39	−0.95^b^	0.348
STAI‐T	34.23 ± 7.09	34.14 ± 6.94	0.056^b^	0.956	34.23 ± 4.91	34.14 ± 5.17	0.055^b^	0.956
BDI‐II^a^	0 (0, 2.75)	1 (0, 6)	813^a^	0.307	1.25 (0, 4.13)	2.5 (0.5, 4.75)	181^a^	0.218
CPS	53 (51, 59.75)	52.5 (48, 59.25)	825^a^	0.392	56.25 (51, 57.63)	53.5 (48, 57.25)	177.5^a^	0.192
PSS‐10	11.57 ± 5.83	11.33 ± 5.66	0.189^b^	0.850	11.57 ± 3.68	11.33 ± 3.83	0.205^b^	0.838
RRS	30 (25, 40.5)	33 (24, 41.25)	902.5^a^	0.852	32.93 ± 5.89	34.24 ± 8.27	−0.599^b^	0.553
GPS	37.30 ± 5.48	38.24 ± 5.21	−0.817^b^	0.416	37.30 ± 3.52	38.24 ± 3.90	−0.832^b^	0.410
ToM	13 (12, 15)	13.5 (12, 15)	841.5^a^	0.468	13.23 ± 1.59	13.55 ± 1.15	−0.755^b^	0.455
PT	15 (14, 17.75)	15 (14, 18)	887.5^a^	0.749	15.46 ± 2.59	15.41 ± 2.11	0.069^b^	0.945
EC	16.98 ± 3.08	16.76 ± 3.56	0.301^b^	0.764	16.98 ± 2.18	16.76 ± 2.59	0.295^b^	0.769
RPHNS‐T	9.36 ± 11.4	6.83 ± 12.02	1.002^b^	0.319	9.36 ± 6.58	6.83 ± 8.82	1.069^b^	0.291

*Note*: Data are reported as the mean ± standard deviation or median (P25, P75).

Abbreviations: BDI‐II, Beck Depression Inventory‐II (Beck et al. 1996); BMI, body mass index; CPS, cooperative personality scale (Xie et al. 2006); EC, empathic concern (Zhang et al. 2010); GPS, group preference scale (Larey and Paulus 1999); PSS‐10, perceived stress scale (Cohen et al. 1983; Huang et al. 2020); PT, perspective taking (Zhang et al. 2010); RPHNS‐T, trustworthiness factor of the Revised Philosophies of Human Nature Scale (Jian and Tang 2006); RRS, ruminative response scale (Han and Yang 2009); STAI‐T, Trait version of the State‐Trait Anxiety Inventory (Spielberger et al. 1999); ToM, theory of mind (Xue et al. 2022).

^a^Mann‒Whitney *U* test.

^b^Independent sample *t‐*test.

### 3.2 Manipulation Check

**FIGURE 4:**
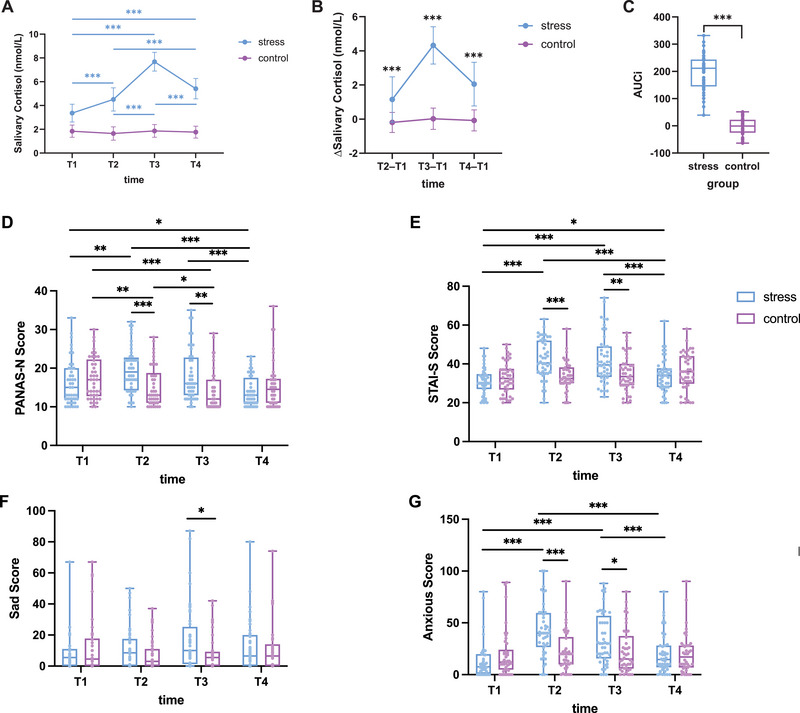
Physiological and psychological responses to acute stress: (A) Salivary cortisol concentrations, (B) the change values of salivary cortisol relative to T1, (C) AUCi for salivary cortisol, (D) negative affect scores, (E) state anxiety scores, (F) sadness dimension scores on the visual analogue scale, and (G) anxiousness dimension scores on the visual analogue scale. Error bars in A and B represent standard deviations. The boxes extend from the 25th to 75th percentiles, with the horizontal line representing the median. The whiskers show the min‐to‐max values. The dots represent individual data points. *p < 0.05, **p < 0.01, ***p < 0.001.

A significant time × group interaction effect was observed for salivary cortisol levels (Wald *χ* ^2^(3) = 544.071, *p* < 0.001). Post hoc analyses revealed significant differences between all time points in the stress group (all *p*s < 0.001; Figure 4A), whereas no significant differences were observed between any time points in the control group (all *p*s > 0.05). Between‐group comparisons based on change values (relative to T1) also showed a significant interaction (Wald *χ* ^2^(2) = 223.45, *p* < 0.001). As shown in Figure 4B, the stress group demonstrated significantly greater change values at T2, T3, and T4 compared to the control group (all *p*s < 0.001). The stress group also exhibited significantly higher cortisol AUCi values than the control group (*t*_(64.089)_ = 17.839, *p* < 0.001; Figure 4C). Collectively, these cortisol findings confirm successful stress induction in the stress group, along with a sustained stress response throughout the experiment, whereas no notable stress response was detected in the control group.

For positive affect (PANAS), a marginally significant time × group interaction was found (Wald *χ* ^2^(3) = 7.722, *p* = 0.052), with no between‐group differences at any time point (all *p*s > 0.05). Detailed GEE model parameter estimates are reported in Table S1, including regression coefficient (*β*), standard error (SE), Wald *χ* ^2^, *p* value, and 95% confidence interval (CI). Significant interactions were also observed for the PANAS negative affect score (Wald *χ* ^2^(3) = 54.322, *p* < 0.001), STAI‐S score (Wald *χ* ^2^(3) = 52.466, *p* < 0.001), VAS‐sad score (Wald *χ* ^2^(3) = 18.843, *p* < 0.001), and VAS‐anxious score (Wald *χ* ^2^(3) = 43.694, *p* < 0.001). Specifically, the participants in the stress group reported higher negative affect (T2: Wald *χ* ^2^(1) = 13.125, *p* < 0.001; T3: Wald *χ* ^2^(1) = 9.496, *p* = 0.002) and higher state anxiety (T2: Wald *χ* ^2^(1) = 18.006, *p* < 0.001; T3: Wald *χ* ^2^(1) = 9.863, *p* = 0.002) relative to the control group (Figure 4D,E). State anxiety in the stress group was also significantly lower at T1 than at later time points (*p*s < 0.05; Figure 4E). Elevated sadness (T3: Wald *χ* ^2^(1) = 6.428, *p* = 0.011; Figure 4F) and anxiousness scores (T2: Wald *χ* ^2^(1) = 19.404, *p* < 0.001; T3: Wald *χ* ^2^(1) = 5.768, *p* = 0.016; Figure 4G) were also observed in the stress group. Despite a significant interaction effect on the VAS‐happy score (Wald *χ* ^2^(3) = 16.898, *p* = 0.001), no between‐group differences were detected at any time point (all *p*s > 0.05). No significant interaction effects were observed for the VAS‐tired or VAS‐withdrawn scores (both *p*s > 0.05). These findings confirmed the successful induction of acute stress in the stress group.

### 3.3 Behavioral Task Performance

A comprehensive summary of the behavioral outcomes for all the experimental tasks is provided in Table S2. No significant between‐group differences were observed for any of the task measures at baseline (all *p*s > 0.05). A preliminary analysis of within‐dyad baseline RT compatibility revealed that not all dyads were closely matched in terms of individual response speed during cooperation, with some showing statistically significant differences (e.g., one dyad in the stress group: 218.41 ± 26.68 ms vs. 200.3 ± 30.04 ms; *t*_(52)_ = 2.342, *p* = 0.023).

#### 3.3.1 Executive Function

**FIGURE 5:**
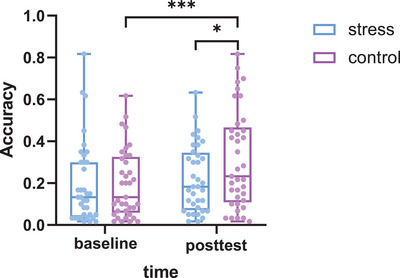
Accuracy in the 3‐back task. The boxes extend from the 25th to 75th percentiles, with the horizontal line representing the median. The whiskers show the min‐to‐max values. The dots represent individual data points. *p < 0.05, ***p < 0.001.

In the 3‐back task, the results revealed a significant time × group interaction (Wald *χ* ^2^(1) = 4.061, *p* = 0.044) for 3‐back accuracy. The post hoc analysis revealed significantly higher accuracy in the control group from baseline to posttest (Wald *χ* ^2^(1) = 12.567, *p* < 0.001) and lower accuracy in the stress group than in the control group at posttest (Wald *χ* ^2^(1) = 3.862, *p* = 0.049) (Figure 5). The main effect of time for 3‐back accuracy was also significant (Wald *χ* ^2^(1) = 9.828, *p* = 0.002). The interaction effect and the main effect of time for the 3‐back RT were not significant (*p*s > 0.05). In the Go/Nogo task, none of the interaction effects reached significance for the IES, Nogo accuracy, or goRT (all *p*s > 0.05). However, a significant main effect of time was observed for goRT (Wald *χ* ^2^(1) = 12.735, *p* < 0.001). Similarly, no significant interactions were detected for the Stroop effect or switching cost (both *p*s > 0.05), though switching cost showed a significant main effect of time (Wald *χ* ^2^(1) = 12.688, *p* < 0.001).

#### 3.3.2 Social Function

In the cooperation task, none of the group × time interaction effects for the three outcome measures were significant (all *p*s > 0.05). However, the main effects of time were all significant (all *p*s < 0.01). No significant interaction effects were observed for any of the subjective ratings measuring participants’ attitudes toward their partners or their subjective experiences during the task, which included partner favorability, trust in the partner, in‐task cooperativeness, in‐task pleasantness, in‐task concentration degree, and shared intention (all *p*s > 0.05).

### 3.4 fNIRS Results

#### 3.4.1 Intrabrain Activation

**FIGURE 6:**
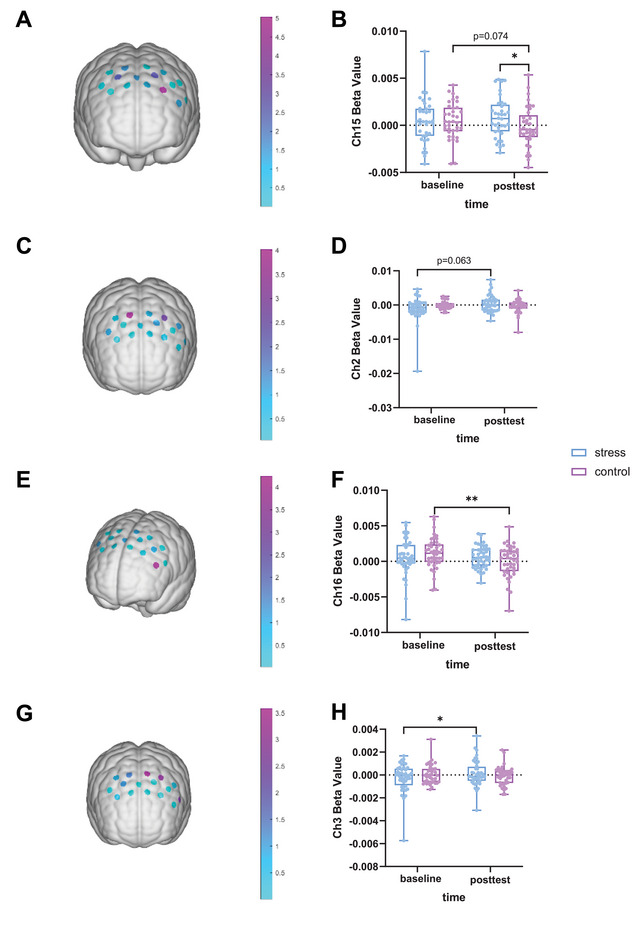
PFC activation results during the 3‐back task (A and B), Stroop task (C and D), switching task (E and F), and cooperation task (G and H) before and after the stress/control manipulations. (A), (C), (E), and (G): Uncorrected statistical maps of Wald *χ* ^2^ for the group × time interaction effect for each channel. (B), (D), (F), and (H): Box and whisker plots depicting beta values. The boxes extend from the 25th to 75th percentiles, with the horizontal line representing the median. The whiskers show the min‐to‐max values. The dots represent individual data points. *p < 0.05, **p < 0.01.

For the 3‐back task, an uncorrected significant time × group interaction was observed for left DLPFC activation (Ch15: Wald *χ* ^2^(1) = 5.027, *p* = 0.025, *p*_FDR_ > 0.05; Figure 6A). Exploratory post hoc analysis suggested significantly higher beta values in the stress group than in the control group at the posttest assessment (Wald *χ* ^2^(1) = 4.036, *p* = 0.045). The participants in the control group showed a marginally significant decrease in Ch15 activation from baseline to the posttest (Wald *χ* ^2^(1) = 3.192, *p* = 0.074; Figure 6B), whereas the stress group showed a nonsignificant increase. No significant interaction effects were detected for channel activation in the Go/Nogo task (all *p*s > 0.05). For the Stroop task, no significant interaction effects were found in predefined ROIs. Exploratory analyses, however, suggested an uncorrected significant interaction effect in the right frontal eye fields (FEF) (Ch2: Wald *χ* ^2^(1) = 4.015, *p* = 0.045; Figure 6C), with the stress group exhibiting a marginally significant increase in Ch2 beta values over time (Wald *χ* ^2^(1) = 3.453, *p* = 0.063; Figure 6D) and the control group showing a decreasing trend. In the task‐switching paradigm, whereas no significant interaction effects were observed within ROIs, exploratory analysis revealed an uncorrected significant interaction for the activation of the left pars triangularis (Ch16: Wald *χ* ^2^(1) = 4.237, *p* = 0.04; Figure 6E), with the control group showing significantly decreased Ch16 beta values from baseline to the posttest (Wald *χ* ^2^(1) = 8.006, *p *= 0.005; Figure 6F), whereas the activation of this region tended to increase in the stress group. During the cooperation task, no significant interactions emerged in the predefined ROIs. Exploratory analysis revealed a marginally significant but uncorrected interaction effect for left FEF activation (Ch3: Wald *χ* ^2^(1) = 3.578, *p* = 0.059; Figure 6G), with the Ch3 beta values increasing significantly from baseline to the posttest in the stress group (Wald *χ* ^2^(1) = 3.927, *p* = 0.048; Figure 6H) and decreasing in the control group.

#### 3.4.2 Interbrain Synchronization

**FIGURE 7:**
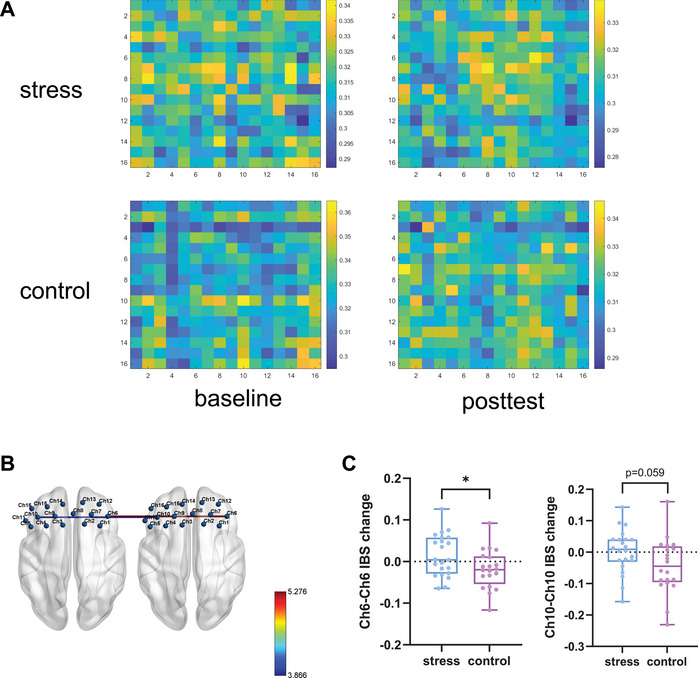
IBS in the cooperation task: (A) group‐averaged IBS matrices. The color bar represents the IBS values. (B) Significant interaction effects for Ch6–Ch6 and Ch10–Ch10. The color bar represents the Wald *χ* ^2^ value. The ball‐and‐stick model was visualized via BrainNet Viewer (Xia et al. 2013). (C) IBS change results. The boxes extend from the 25th to 75th percentiles, with the horizontal line representing the median. The whiskers show the min‐to‐max values. The dots represent individual data points. *p < 0.05.

Figure 7A shows the group‐averaged IBS results during the cooperation task. For the symmetric IBS, significant group × time interaction effects were observed for Ch6–Ch6 (Wald *χ* ^2^(1) = 5.276, *p* = 0.022) and Ch10–Ch10 (Wald *χ* ^2^(1) = 3.866, *p* = 0.049) (Figure 7B). Following FDR correction, these effects were marginally significant (both *p*_FDR_ > 0.05). IBS in channels Ch6–Ch6 and Ch10–Ch10 was significantly above zero during both baseline and posttest sessions across groups (all *p*s < 0.001), indicating the presence of IBS in these regions. The baseline IBS values for Ch6–Ch6 and Ch10–Ch10 showed borderline significant differences between the groups (*p* = 0.053 and *p* = 0.052, respectively). The change values (posttest–baseline) were calculated to account for baseline variability and conformed to a normal distribution. Independent sample *t*‐tests revealed that the IBS change values were significantly greater in the stress group than in the control group (Ch6–Ch6: *t*_(40)_ = 2.233, *p* = 0.031; Ch10–Ch10: *t*_(40)_ = 1.94, *p* = 0.059) (Figure 7C).

### 3.5 Correlation Analysis of Executive and Social Function

**FIGURE 8:**
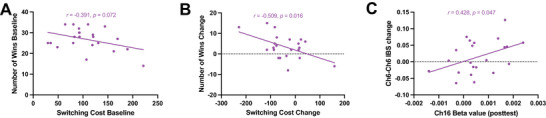
Person correlation analyses of the stress group: (A) relationship between the switching cost and the number of wins at baseline, (B) relationship between the change in switching cost and the change in the number of wins, (C) relationship between Ch16 beta values in the posttest during the task‐switching paradigm and changes in Ch6–Ch6 IBS values during cooperation.

In the stress group, the dyad‐level switching cost (i.e., the mean value of the two participants) was negatively associated with the number of wins at baseline at a marginally significant level (*r* = −0.391, *p* = 0.072) (Figure 8A). The change in switching cost was negatively correlated with the change in the number of wins (*r* = −0.509, *p* = 0.016) (Figure 8B), an association that was not observed in the control group. No significant associations were observed between the number of wins and any other primary behavioral outcomes—specifically, 3‐back accuracy in the 3‐back task, IES in the Go/Nogo task, or the Stroop effect in the Stroop task—either in baseline values or the change values (all *p*s > 0.05). At the neural level, beta values in Ch16 during the posttest task‐switching paradigm showed a positive correlation with Ch6–Ch6 IBS change values in the cooperation task (*r* = 0.428, *p* = 0.047) (Figure 8C), but this correlation was not detected in the control group. No significant associations were observed between 3‐back/Stroop task‐related activation and cooperative IBS (both *p*s > 0.05). Similarly, no significant correlations were detected between any of the neural indicators and the behavioral performance measures (all *p*s > 0.05).

## 4 Discussion

Conflicting evidence on the neurobehavioral consequences of acute psychosocial stress on executive function and social cooperation has been presented in the literature, with a limited understanding of the relationships between these factors. In this preliminary study, we sought to begin addressing this gap through a multimodal investigation combining behavioral paradigms (3‐back, Go/Nogo, Stroop, task‐switching, and cooperation tasks) and neuroimaging (activation and IBS). The comparable basic characteristics between the stress and control groups suggest that preexisting individual differences were unlikely to confound the experimental results. Furthermore, the lack of group differences in subjective attitudes toward the cooperation task suggests that the observed effects are not attributable to motivational or evaluative bias. Our exploratory analyses yielded several observations worthy of further attention. Acute stress selectively impaired the learning‐related improvement in 3‐back working memory performance observed in the control group, although it did not significantly affect behavioral performance in the other tasks. The brain activation findings are predominantly exploratory and should be interpreted as preliminary evidence. The findings suggested that stress may be associated with a blockade of learning‐related neural efficiency gains. Furthermore, our exploratory analysis revealed trends in neural activation that may be indicative of compensatory mechanisms in the PFC in the stress group. Hyperscanning data also revealed that stress resulted in a greater IBS increase in the PFC during cooperative interactions, suggesting possible compensatory interbrain hypercoupling in stress states and extending the exploration of neural mechanisms to the two‐person context. Notably, in the stress group, switching cost was negatively correlated with the number of wins, suggesting that better cognitive flexibility appears to be associated with more successful cooperation. Moreover, brain activation during the task‐switching paradigm was positively correlated with IBS changes during cooperation, tentatively supporting a potential link between cognitive and social processes under stress. To our knowledge, this work offers an initial integrated examination of how acute stress influences social and cognitive processes across individual and interactive contexts.

In contrast to our hypothesis, acute stress had no significant effect on performance in tasks measuring inhibition control or cognitive flexibility. However, a significant effect was observed specifically in the high‐load 3‐back working memory task. This dissociation may be explained by differences in the cognitive load imposed by each task. Previous research indicates that acute stress tends to exert more pronounced disruptive effects on tasks requiring high cognitive demand (Oei et al. 2006; Jiang and Rau 2017b). In the present study, the 3‐back task constituted a high‐workload condition (Gaertner et al. 2014; Liang et al. 2024), whereas the Go/Nogo, Stroop, and switching tasks may be considered cognitively less demanding (Song et al. 2020). These lower load tasks place relatively lower demands on executive control and may therefore be less sensitive to the subtle performance disruptions caused by acute stress (Schoofs et al. 2009). This interpretation is further supported by the performance trajectories of the two groups. The control group exhibited improved 3‐back accuracy from the baseline to posttest, suggesting a practice‐related learning effect. In contrast, the stress group showed no such improvement, resulting in significantly lower accuracy relative to the control group after stress induction. This pattern indicates that acute stress impeded the typical learning effect in the highly demanding task, highlighting the detrimental impact of stress on adaptive working memory processes. These findings are consistent with earlier reports of stress‐induced impairment under high cognitive load (Schoofs et al. 2008; Gaertner et al. 2014).

The hypothesis that DLPFC activity related to these processes decreases under stress was not validated. In the exploratory analysis of the 3‐back task, a pattern was observed wherein the control group exhibited a reduction in left DLPFC (Ch15) activation from the baseline to posttest. As a key node within the ECN (Hermans et al. 2014; van Oort et al. 2017), the DLPFC is central to working memory processes (Curtis and D'Esposito 2003; Barbey et al. 2013). This reduced activity is consistent with the possibility that repeated task exposure increased neural efficiency, enabling maintained or improved performance with fewer cognitive resources. In contrast, the stress group showed no such adaptive change and maintained a consistently high level of activation. This preliminary pattern is compatible with the interpretation that acute stress may disrupt the natural optimization of neural circuitry through learning (i.e., learning‐related gains in neural efficiency), potentially necessitating continued reliance on heightened resource mobilization to maintain cognitive performance.

Similarly, during the task‐switching paradigm, the exploratory analyses demonstrated significantly reduced activation in the left pars triangularis (Ch16) from the baseline to posttest in the control group, whereas an increasing trend of neural activation was observed in the stress group. As a subregion of the inferior frontal gyrus (IFG), the pars triangularis is critically involved in goal‐directed cognitive control. The IFG has consistently been implicated in task‐switching demands in prior neuroimaging studies, and lesions in this region have been associated with increased switching costs (Aron et al. 2004; Rodriguez‐Nieto et al. 2022). Notably, this neurophysiological dissociation between groups was observed despite the absence of differences in the switching cost. This observation could be interpreted as suggesting that participants in the stress group may have needed to sustain neural engagement in the IFG to maintain task performance comparable to that of the control group, a pattern again consistent with a potential blockade of learning‐related neural efficiency gains under stress. In contrast, the reduced neural activation in the control group reflects the abovementioned practice‐driven gains in neural efficiency.

During the Stroop task, exploratory analysis indicated a trend toward increased activation in the right FEF (Ch2) from the baseline to postintervention in the stress group. The FEF, a critical node in the ECN (Hermans et al. 2014; van Oort et al. 2017), is involved in spatial attention allocation and top‐down goal‐directed attention control (Bradley et al. 2022). The FEF supports functions such as maintaining and manipulating information in the context of goal‐oriented behavior—processes that are integral to resolving interference in the Stroop task. No significant difference in the Stroop effect was observed between groups. Notably, increased brain activation in the right FEF was observed in the stress group, suggesting that stressed participants required more cognitive resources to maintain performance. In contrast, a decreasing trend in activation from the baseline to postintervention was observed in the control group, which was consistent with the findings obtained using the other tasks. However, in the Go/Nogo task, stress failed to significantly modulate either behavioral performance or neural activation. This null finding may be attributed to the relative simplicity of the task (i.e., low cognitive demand), which likely induced a ceiling effect, but it also underscores the preliminary nature of the neural patterns observed in other tasks.

According to the biphasic–reciprocal model, acute stress causes a shift in brain activity from the ECN to the salience network (Hermans et al. 2014; Arnsten 2015). More specifically, acute stress is often assumed to result in reduced DLPFC activity (Qin et al. 2009), whereas stimulating the DLPFC counteracts the effect of stress on working memory (Bogdanov and Schwabe 2016). However, moderate stressors have been linked to increased recruitment of PFC regions, a compensatory mechanism that correlates with preserved task performance (Porcelli et al. 2008; Mandrick et al. 2016; Causse et al. 2022; Schwartz et al. 2025). This phenomenon, termed compensatory neural activation, reflects the adaptive response of the brain to stress, with additional neural resources recruited to sustain function. Theoretical models such as the processing efficiency theory and the cognitive–energetical framework provide conceptual foundations for understanding how stress modulates performance (Eysenck and Calvo 1992; Hockey 1997). According to these models, compensatory efforts, such as increased prefrontal engagement, can temporarily offset stress‐related performance decreases. However, this compensation comes at a cognitive cost, as the recruitment of additional neural resources reduces neural efficiency. The exploratory neural patterns observed in the present study may be interpreted as aligning with this latter perspective, suggesting that the stress‐induced trends of increased prefrontal activity could be indicative of potential compensatory engagement aimed at supporting cognitive function. However, it is critical to emphasize that, given the preliminary nature of our neural data, this remains a speculative interpretation. Furthermore, on the basis of existing literature, such compensation might fail under conditions of inefficient neural mechanisms, resource depletion over extended periods of stress, or exposure to more severe stress.

Our findings—specifically, the maintenance of cooperative performance under acute stress, coupled with exploratory neural trends suggesting increased investment—invite a nuanced discussion within the adaptive framework of “tend‐and‐befriend” stress response pattern in males. The pattern observed here may be more precisely characterized as a “stress‐induced investment to maintain social bonds,” rather than a full “tend‐and‐befriend” response marked by increased prosociality. Behaviorally, no differences were observed between groups. At the neurophysiological level, the exploratory analyses suggested a pattern wherein the stress group exhibited increased activation in the left FEF (Ch3), whereas the control group showed reduced activation in the same region. The activation of the left FEF during the cooperative button‐pressing task aligns with prior neuroimaging findings that this type of task requires participants to actively attend to the visual stimuli presented on the screen (Liu et al. 2023). This trend of increased neural activation under stress may be compatible with the compensatory mechanism discussed earlier. Moreover, the results revealed increased IBS in the right (Ch6–Ch6) and left (Ch10–Ch10) DLPFC in both groups. This finding aligns with prior research showing commonly increased IBS in the left and right DLPFC during cooperative tasks (Cheng et al. 2015; Reindl et al. 2018; Xue et al. 2018; Miller et al. 2019; Hu et al. 2021). The DLPFC is a critical region for sensorimotor synchronization and theory of mind (Feng et al. 2020; Liu et al. 2023; Niu et al. 2024). Sensorimotor synchronization coordinates group actions through the adjustment of individual behaviors, whereas theory of mind allows individuals to infer the mental states of other individuals, both of which enhance cooperative performance. Critically, our exploratory data suggested that stress was associated with a greater increase in IBS during cooperation compared to the control condition. One plausible explanation is that this elevated IBS may be partly attributable to the shared experience of stress induction within each dyad. This common stressful context could promote emotional alignment or enhance coordinative motivation, resulting in heightened neural coupling. However, in contrast to the earlier proposal that a lower IBS in the absence of behavioral differences may reflect streamlined or efficient neural collaboration (Hu et al. 2025), the observed trend of heightened IBS under stress could be interpreted as suggesting a compensatory effect rather than efficient neural collaboration. We cautiously propose that this pattern might represent an extension of neural compensation to the interpersonal domain. As noted previously, baseline IBS trends and the preliminary nature of these neural data necessitate caution in this interpretation. A prior study investigating young women under acute stress revealed increased prefrontal IBS and reduced RT differences between participants over time, supporting the idea that stress strengthens the “tend‐and‐befriend” response in females during cooperation (Zhang et al. 2021). In contrast, although our study in males did not show enhanced prosocial behavior, the co‐occurrence of maintained cooperative performance with trends suggesting increased neural investment could reflect a related adaptive principle: Stress may prompt increased investment to sustain social bonds and joint performance. This perspective refines the application of the “tend‐and‐befriend” model, highlighting that under acute stress, more investment may be directed toward preserving existing social function, not necessarily enhancing it.

However, an important alternative interpretation of these neural patterns must be considered. The observed increases in PFC activation and interbrain synchrony could alternatively reflect a general, task‐nonspecific “stress‐state” signature, such as heightened physiological arousal, increased task‐independent worry, or the overall cognitive load of being under stress. The fact that elevated activity was observed across multiple distinct tasks lends support to this view, suggesting a pervasive influence of the stress condition itself on neural activity. In considering these perspectives, several aspects of our exploratory data may tentatively favor—though not conclusively prove—a task‐related compensatory interpretation, without ruling out the influence of a general stress state. First, the neural increases, while preliminary, were not entirely diffuse; they occurred in regions with established relevance to the specific cognitive demands of each task (e.g., DLPFC in working memory, IFG in task‐switching). Second, and perhaps more critically, this increased neural engagement co‐occurred with the preservation of behavioral performance at the group level. A pure “stress‐state” or “interference” account might predict performance degradation. The conjunction of stable performance and elevated, regionally plausible neural activity could be viewed as consistent with the possibility that additional resources were engaged to help maintain function under stress—an interpretation aligned with compensatory models. Crucially, these interpretations are not mutually exclusive and may represent overlapping processes. The present data cannot definitively dissociate task‐specific compensatory effort from more general stress‐related neural load. Therefore, we present the compensatory account as a plausible, though provisional, framework for understanding our specific pattern of results, emphasizing that it remains a working hypothesis. Future studies with designs aimed at dissociating these mechanisms will be essential to clarify their respective contributions.

We observed a positive correlation between cognitive flexibility and cooperative performance, which aligns partially with our initial hypothesis. Building on previous work suggesting a role for executive control in social interactions (Guo, Huang, et al. 2023; Wang et al. 2024; Zhang et al. 2024), this study provides preliminary evidence linking cognitive flexibility to cooperative outcomes in a stress context. One possible explanation is that cognitive flexibility may enhance an individual's ability to shift between self‐ and other‐oriented perspectives, thereby supporting dynamic behavioral adjustments that facilitate interpersonal coordination in tasks such as the cooperative button‐pressing task used here. At the neural level, we found that prefrontal activation during task‐switching was positively correlated with IBS during cooperation, suggesting a tentative link across neural and behavioral domains. Together, these correlations, particularly the single, uncorrected correlation between task‐switching activation and cooperation IBS, provide a limited but intriguing basis for further inquiry. They are consistent with a broader perspective in which executive and social functions are not fully independent but may interact at both behavioral and neural levels, particularly under acute stress. To integrate these preliminary observations and generate a concrete target for future research, we tentatively propose the “Executive–Social Function Coupling Hypothesis under Stress” as a speculative, heuristic framework. This framework is motivated by the tendency of existing theories to treat executive and social processes separately, despite indications of their interrelation (Zhang et al. 2024). We explicitly emphasize that this hypothesis is derived directly from our exploratory findings and remains highly provisional. Its primary value lies in offering a testable model that requires independent replication and validation through dedicated studies.

A key finding—and a challenge to a straightforward compensation hypothesis—was the lack of a significant correlation between the neural indicators and behavioral measures. If heightened neural activity solely reflected effective compensation, a positive correlation might be expected. This null finding necessitates a more nuanced view, suggesting that the observed group‐level neural engagement may represent a heterogenous process that is not uniformly efficient at the individual level. Several non‐mutually exclusive factors may explain this dissociation and refine the compensation hypothesis. First, the demanding nature of the tasks under stress likely engages a broader compensatory network. Thus, although the neural measures captured here are relevant, they may be necessary but not sufficient to fully explain behavioral outcomes, with additional unmeasured neural or cognitive resources contributing to performance. Second, increased activation might sometimes reflect the high neural cost or effort of maintaining performance (i.e., the process of compensation), rather than its guaranteed success. This cost can vary independently of behavioral output, weakening correlations. Third, individual differences in cognitive strategy or neural efficiency may have obscured correlations. For some participants, higher neural activation might facilitate performance, whereas for others, it may indicate struggling with minimal gain. Fourth, temporal dissociations between hemodynamic responses and behavioral outputs under stress may further obscure direct links. Finally, this study may have been underpowered to detect subtle neural–behavioral relationships. Therefore, although the co‐occurrence of increased neural activity and stable performance at the group level may be consistent with a potential compensatory response, the null correlation highlights the complexity of this process. It suggests that “compensation” under stress is not a monolithic or uniformly efficient mechanism, but a variable process whose neural signatures can reflect both successful adaptation and costly effort. This distinction offers a critical framework for future research.

The results of this study have several critical implications. Theoretically, our exploratory findings reveal patterns consistent with potential compensatory neural mechanisms that may support stable task performance following moderate stress exposure and suggest the possibility that this mechanism could extend to dyadic brain networks. Our work provides initial evidence pertinent to a “stress‐induced investment to maintain social bonds and joint performance” response pattern in males, rather than demonstrating a full “tend‐and‐befriend” response. Our finding offers initial evidence linking executive control processes to social interaction dynamics under acute stress, at both behavioral and neural levels. To integrate these preliminary observations, we propose the “Executive–Social Function Coupling Hypothesis under Stress” as a purely exploratory and speculative framework to be tested and refined in future studies. In terms of the clinical implications, because impairments in executive function and social interaction are established risk factors for psychopathology, the patterns observed here may inform future investigations into the mechanistic pathways linking acute stress to psychiatric disorders, potentially aiding the refinement of theoretical models of related neuropsychiatric conditions.

Despite these novel findings, this study has several limitations. First, the sample was limited to a homogeneous group of healthy male college students, which may restrict the generalizability of the findings. Further studies are warranted to determine whether these effects are generalizable to females, nonstudent populations, and clinical groups. Second, limited sensitivity was observed in the Go/Nogo task employed to measure response inhibition, and we failed to detect behavioral or neural differences among our participants. Future studies should prioritize the use of more discriminative paradigms. Third, the dyads were not explicitly matched on personality dimensions or baseline response speed. Although group‐level comparisons showed no significant differences in individual characteristics between experimental conditions, the lack of within‐dyad homogeneity in questionnaire‐derived traits may have introduced unaccounted variance in cooperation (Balconi et al. 2017; Liu et al. 2023). Additionally, results indicated that some dyads also differed significantly in their baseline RT during cooperation. This inherent variability in both trait composition and response speed within dyads could have introduced additional unsystematic variance into the measurement of cooperative performance. Future studies should consider controlling for interpersonal similarity in both psychological traits and baseline task performance when forming dyads to better isolate the effects of experimental manipulations on cooperative behavior. Fourth, the strategic implications of the cooperation task were not fully explored. The task inherently encourages coordination over speed: success depended on relative—not absolute—timing between partners, rewarding delayed but synchronized responses over rapid but uncoordinated ones. Although no explicit strategy was provided, the reward structure naturally prompted participants to anticipate and match their partner's timing. Investigating how explicit strategic instructions influence neural and behavioral synchrony represents a promising direction for future research. Fifth, as an initial investigation in this area, the brain activation findings are presented as exploratory. The observed patterns did not withstand strict FDR correction—a limitation that underscores the study's limited statistical power to detect subtle effects and to reliably survive stringent multiple comparisons. However, they offer valuable initial insights and point to specific neural correlates for future research. Therefore, these results should be interpreted as hypothesis‐generating, highlighting promising neural patterns that require confirmation in future, larger scale studies. More broadly, the study was likely underpowered to detect subtle brain‐behavior relationships, which typically exhibit small effect sizes and necessitate larger, confirmatory samples for reliable detection. Sixth, it should be noted that baseline IBS values showed borderline significant differences between groups. Although our primary analysis used change scores to control for these initial values and to focus on stress‐induced changes, the pre‐existing differences remain a consideration when interpreting the magnitude of the observed effects. Finally, although we measured salivary cortisol, we did not assay other hormonally relevant markers such as testosterone, which may exhibit diurnal fluctuations and influence stress responses. Future studies should incorporate multisystem hormonal assessments to more comprehensively evaluate neuroendocrine contributors to stress dynamics.

## 5 Conclusions

This study provides a preliminary systematic investigation of the effects of acute stress on changes in social and cognitive functions among both individuals and dyad pairs. The experiments and analyses yielded three main observations. First, TSST‐G‐induced stress selectively impaired adaptive working memory performance but did not impact response inhibition, interference control, cognitive flexibility, or cooperative performance. Second, exploratory observations revealed trends of increased PFC activation across distinct tasks and elevated IBS during dyadic cooperation following stress exposure. These neural patterns are compatible with, but do not confirm, potential compensatory processes that may contribute to preserved performance, and they align with a conceptualization of “stress‐induced investment to maintain social bonds” in males. Third, the observed neural and behavioral correlations offer preliminary evidence contributing to an emerging picture of executive‐social integration under stress. We frame these exploratory findings within the tentatively proposed “Executive–Social Function Coupling Hypothesis under Stress” as a heuristic model for future research. Collectively, these results offer initial insights and conceptual groundwork for understanding stress‐modulated cognitive‒social interactions.

## Author Contributions

**Zhihua Guo**: conceptualization, formal analysis, investigation, methodology, writing – original draft, writing – review and editing. **Yue Gong**: investigation, writing – original draft. **Liu Yang**: investigation, writing – original draft. **Yushan Li**: investigation, methodology. **Rui Qiu**: investigation, methodology. **Xia Zhu**: conceptualization, funding acquisition, project administration, supervision, writing – review and editing.

## Funding

This work was supported by the Key Logistics Science and Technology Project (Grant/Award Number: AKJWS221J001).

## Conflicts of Interest

The authors declare no conflicts of interest.

## Supporting information



Supplementary Table: brb371214‐sup‐0001‐TableS1‐S2.docx

## Data Availability

The data that supported the present study are available from the corresponding author upon reasonable request.
