# Supplementary material for: Acute Stress Impacts Executive‐Social Function: Evidence From Prefrontal Activation and fNIRS‐Based Hyperscanning
Source: Brain Behav. 2026 Jan 15;16(1):e71214. doi: 10.1002/brb3.71214 (PMC12808932; doi:10.1002/brb3.71214)
Supplement: Supplementary file 1 — Supplementary Table: brb371214‐sup‐0001‐TableS1‐S2.docx [file BRB3-16-e71214-s001.docx]

Table 1 Parameter estimates from the generalized estimating equation model for statistically significant interactions

| Variable | *β* | SE | Wald χ^2^ | *p* | 95% CI |
| --- | --- | --- | --- | --- | --- |
| Salivary cortisol | | | | | |
| Group (stress) × Time (1st) | -2.13 | 0.2135 | 99.496 | <0.001 | (-2.548, -1.711) |
| Group (stress) × Time (2nd) | -0.786 | 0.2084 | 14.234 | <0.001 | (-1.195, -0.378) |
| Group (stress) × Time (3rd) | 2.167 | 0.1979 | 119.92 | <0.001 | (1.779, 2.555) |
| ∆Salivary cortisol (relative to T1) |  |  |  |  |  |
| Group (stress) × Time (1st) | -0.786 | 0.2084 | 14.234 | <0.001 | (-1.195, -0.378) |
| Group (stress) × Time (2nd) | 2.167 | 0.1979 | 119.92 | <0.001 | (1.779, 2.555) |
| Positive affect | | | | | |
| Group (stress) × Time (1st) | -2.933 | 1.245 | 5.554 | 0.018 | (-5.372, -0.494) |
| Group (stress) × Time (2nd) | -2.995 | 1.254 | 5.706 | 0.017 | (-5.452, -0.538) |
| Group (stress) × Time (3rd) | -2.833 | 1.122 | 6.373 | 0.012 | (-5.033, -0.634) |
| Negative affect | | | | | |
| Group (stress) × Time (1st) | 0.529 | 1.179 | 0.202 | 0.653 | (-1.781, 2.84) |
| Group (stress) × Time (2nd) | 5.872 | 1.032 | 32.373 | <0.001 | (3.849, 7.895) |
| Group (stress) × Time (3rd) | 5.562 | 0.906 | 37.704 | <0.001 | (3.786, 7.337) |
| STAI-S | | | | | |
| Group (stress) × Time (1st) | -0.438 | 1.699 | 0.067 | 0.796 | (-3.769, 2.892) |
| Group (stress) × Time (2nd) | 9.841 | 2.117 | 21.601 | <0.001 | (5.691, 13.991) |
| Group (stress) × Time (3rd) | 9.022 | 2.007 | 20.208 | <0.001 | (5.088, 12.955) |
| VAS-happy | | | | | |
| Group (stress) × Time (1st) | -6.57 | 4.139 | 2.52 | 0.112 | (-14.683, 1.542) |
| Group (stress) × Time (2nd) | -13.723 | 4.566 | 9.034 | 0.003 | (-22.672, -4.774) |
| Group (stress) × Time (3rd) | -17.134 | 4.703 | 13.272 | <0.001 | (-26.352, -7.916) |
| VAS-sad | | | | | |
| Group (stress) × Time (1st) | -4.399 | 3.326 | 1.75 | 0.186 | (-10.918, 2.119) |
| Group (stress) × Time (2nd) | 3.135 | 3.042 | 1.062 | 0.303 | (-2.828, 9.098) |
| Group (stress) × Time (3rd) | 7.904 | 2.721 | 8.434 | 0.004 | (2.569, 13.238) |
| VAS-anxious | | | | | |
| Group (stress) × Time (1st) | -5.876 | 4.856 | 1.464 | 0.226 | (-15.394, 3.643) |
| Group (stress) × Time (2nd) | 20.884 | 4.819 | 18.783 | <0.001 | (11.44, 30.329) |
| Group (stress) × Time (3rd) | 12.148 | 4.338 | 7.843 | 0.005 | (3.646, 20.65) |
| 3-back accuracy | | | | | |
| Group (stress) × Time (baseline) | 0.092 | 0.046 | 4.061 | 0.044 | (0.003, 0.181) |
| 3-back Ch15 activation | | | | | |
| Group (stress) × Time (baseline) | -0.001 | 0.0005 | 5.027 | 0.025 | (-0.002, 0) |
| Stroop Ch2 activation | | | | | |
| Group (stress) × Time (baseline) | -0.002 | 0.0008 | 4.015 | 0.045 | (-0.003, -3.581E-5) |
| Switching Ch16 activation | | | | | |
| Group (stress) × Time (baseline) | -0.001 | 0.0006 | 4.237 | 0.04 | (-0.002, -5.592E-5) |
| Cooperation Ch3 activation | | | | | |
| Group (stress) × Time (baseline) | -0.001 | 0.0003 | 3.578 | 0.059 | (-0.001, 1.873E-5) |
| Cooperation Ch6-Ch6 IBS | | | | | |
| Group (stress) × Time (baseline) | -0.034 | 0.015 | 5.276 | 0.022 | (-0.062, -0.005) |
| Cooperation Ch10-Ch10 IBS | | | | | |
| Group (stress) × Time (baseline) | -0.047 | 0.024 | 3.866 | 0.049 | (-0.094, 0) |

Table 2 Behavioral performance outcomes for all tasks

| Task measures | Stress group | | Control group | |
| --- | --- | --- | --- | --- |
|  | baseline | posttest | baseline | posttest |
| 3-back task | | | | |
| 3-back accuracy | 0.20±0.20 | 0.22±0.16 | 0.20±0.16 | 0.31±0.23 |
| 3-back RT (ms) | 438.46±45.08 | 440.09±54.63 | 440.52±47.66 | 436.30±42.91 |
| Go/nogo task | | | | |
| IES (ms) | 410.26±124.83 | 409.70±325.70 | 406.66±122.48 | 358.65±73.65 |
| goRT (ms) | 373.60±63.97 | 348.47±70.86 | 369.57±73.13 | 341.97±53.35 |
| Nogo accuracy | 0.91±0.08 | 0.90±0.17 | 0.92±0.08 | 0.93±0.07 |
| Colour-word Stroop task | | | | |
| Stroop effect (ms) | 39.42±44.97 | 36.87±36.20 | 31.77±49.82 | 48.40±39.99 |
| Task-switching paradigm | | | | |
| Switching cost (ms) | 108.41±70.26 | 67.58±83.82 | 81.97±67.49 | 49.19±71.82 |
| Cooperation task | | | | |
| Number of wins | 26.73±5.36 | 30.18±2.70 | 27.81±4.26 | 29.05±3.43 |
| Absolute value of the average RT difference of the dyad (ms) | 26.80±4.36 | 24.52±4.78 | 26.57±5.15 | 23.12±4.74 |
| Sum of the average RTs of the dyad (ms) | 479.50±97.41 | 443.34±29.55 | 470.83±54.82 | 438.36±44.36 |

Note: Data are reported as the mean ± standard deviation. RT, response time; IES, inverse efficiency score; goRT, mean RT in the correct go trials.
